# Combining transposon mutagenesis and reporter genes to identify novel regulators of the *topA* promoter in *Streptomyces*

**DOI:** 10.1186/s12934-021-01590-7

**Published:** 2021-05-13

**Authors:** Martyna Gongerowska-Jac, Marcin Jan Szafran, Dagmara Jakimowicz

**Affiliations:** Faculty of Biotechnology, University of Wroclaw, Wroclaw, Poland

**Keywords:** *Streptomyces*, Transcription regulation, *Lux* reporter gene, Transposon mutagenesis, TopA

## Abstract

**Background:**

Identifying the regulatory factors that control transcriptional activity is a major challenge of gene expression studies. Here, we describe the application of a novel approach for in vivo identification of regulatory proteins that may directly or indirectly control the transcription of a promoter of interest in *Streptomyces*.

**Results:**

A method based on the combination of Tn5 minitransposon-driven random mutagenesis and *lux* reporter genes was applied for the first time for the *Streptomyces* genus. As a proof of concept, we studied the *topA* supercoiling-sensitive promoter, whose activity is dependent on unknown regulatory factors. We found that the *sco4804* gene product positively influences *topA* transcription in *S. coelicolor*, demonstrating SCO4804 as a novel player in the control of chromosome topology in these bacteria.

**Conclusions:**

Our approach allows the identification of novel *Streptomyces* regulators that may be critical for the regulation of gene expression in these antibiotic-producing bacteria.

**Supplementary Information:**

The online version contains supplementary material available at 10.1186/s12934-021-01590-7.

## Background

Prokaryotic gene expression is a process that is adjusted to the growth phase and to the changes in environmental conditions. As bacterial gene expression is predominantly regulated at the transcriptional level, bacterial genomes encode numerous proteins that control transcription initiation. Among them, the key players are DNA-binding proteins such as sigma factors, which determine promoter recognition by RNA polymerase (RNAP), as well as other transcription factors (TFs), acting as repressors or activators, which may affect the binding of RNAP to a promoter [6, 39]. However, non-DNA binding proteins such as anti-sigma factors, proteases and other proteins can also control the accessibility of direct regulators to the DNA, thus acting indirectly and playing a critical role in transcriptional regulation. Therefore, the identification of all the components of a regulatory system is a challenging task.

Most often, studies on the regulation of gene expression have been limited to searching for promoters bound and controlled by certain regulatory proteins [8, 31]. To date, several powerful methods for the determination of the DNA-binding sites of known TFs and the genes regulated by them have been developed, such as SELEX, ChIP-chip, ChIP-seq and RNA-seq [5, 30, 33, 45, 78]. All these tools aim to identify all the putative targets of a certain regulatory protein [2]. However, on the other hand, there are only limited strategies to identify TFs of a given promoter of interest [71]. Currently available techniques to identify TFs that bind specific regions include a modified bacterial one-hybrid reporter system [25] and in vitro DNA capture strategies [7, 49, 50, 68]. To search for gene expression regulators in vivo, the combination of random transposon mutagenesis with reporter genes (predominantly *lacZ* or antibiotic resistance cassettes) was successfully developed. This strategy has been applied in a number of bacterial species (*Pseudomonas chlororaphis, Proteus mirabilis, Staphylococcus aureus* and *Vibrio cholerae*) [7, 40–42, 67]. An approach based on the combination of random mutant library construction and the *lux* reporter gene was used for the identification of regulatory proteins of the *lecA* in *Pseudomonas aeruginosa* [16] and the *acs* gene in *Escherichia coli* [2]. Such approaches may be highly beneficial for the identification of global regulatory factors and the dissection of complex regulatory networks, such as those controlling secondary metabolite synthesis in *Streptomyces.*

*Streptomyces* are soil-dwelling bacteria that undergo morphological differentiation, which encompasses vegetative growth and sporulation [20]. They are used as producers of numerous biologically active secondary metabolites, such as antibiotics (approximately 60% of the world’s natural antibiotics are *Streptomyces*-obtained), immunosuppressants and cytostatics [11]. The pathways for the synthesis of secondary metabolites are encoded by gene clusters that are activated only at specific growth phases or physiological conditions [22, 66]. Thus, the production of secondary metabolites is tightly controlled by complex regulatory systems, many of which remain uncharacterized. In silico predictions revealed that the genome of any *Streptomyces* species may encode up to 1100 transcriptional regulators [58], a large fraction of which fall into one of the two main clades: cluster situated regulators (CSRs) and pleiotropic/global regulators [32, 38, 44, 79]. CSRs are regulatory proteins (such as ActII-orf4, RedD and CdaR or TetR, LacI, MerR, and LuxR family regulators [12, 36, 44, 48, 74, 76, 79]) that are usually situated in secondary metabolite biosynthetic gene clusters and directly control the expression of their nearby genes, while pleiotropic regulators (e.g., AdpA [77, 82]), AfsR [21, 28], BldD [15], and DasR [56] are scattered throughout the chromosome and positioned distantly from the genes they regulate. While the identification of CSRs is relatively straightforward, the identification of global regulators that control a particular gene of interest may be challenging [27]. A deep understanding of all aspects of *Streptomyces* gene expression, particularly transcription, is crucial to better exploit these bacteria as producers of widely used compounds.

Notably, in *Streptomyces,* similar to other studied bacteria (*Streptococcus pneumoniae, Haemophilus influenzae, E. coli, Salmonella enterica*), DNA supercoiling also plays a role in global gene regulation by directly affecting the transcriptional activity of a number of promoters [18, 19, 23, 53, 59, 65, 73]. In *Streptomyces,* chromosome supercoiling is a global regulatory factor that controls the transcription of 3–7% of genes [65]. Proper DNA supercoiling in the cell is controlled by a set of enzymes called topoisomerases. The opposing activities of topoisomerase I (TopA), which removes negative supercoils, and gyrase, which can introduce negative supercoils, maintain topological homeostasis in bacterial cells [10]. Inhibition of topoisomerase activity or alteration of their level leads to changes in chromosome topology and affects DNA transactions, including replication and transcription. One of the most important mechanisms that maintains the balance of topoisomerases activity is transcriptional control of their cellular level [46, 69]. In contrast to many model bacterial species, TopA is the only type I topoisomerase in *S. coelicolor;* thus, it is essential and must be precisely regulated to maintain the proper level of chromosomal supercoiling [64]. TopA depletion in *Streptomyces* causes severe growth retardation, including increased DNA supercoiling and altered gene expression, including the expression of secondary metabolite genes [17, 62]. As in other bacteria, in *S. coelicolor,* the TopA level is predominantly regulated by the transcriptional control of the *topA* gene [1, 19, 64, 69]. In *S. coelicolor*, transcription of *topA* is driven from at least two promoters, with equal contributions of both promoters during both vegetative growth and spore production. The *p1* promoter was shown to be supercoiling sensitive, which corroborates the shortened distance between motifs – 10 and – 35 [64]. On the other hand, the comparison of the *p2* promoter to other known promoter sequences did not identify any known recognition site for sigma factors or other transcriptional regulators. Moreover, apart from transcriptional regulation, no other mechanism regulating TopA activity has been described in *Streptomyces*.

Here, to identify regulators of the *topA* promoter in *S. coelicolor,* we used an approach based on Tn5 minitransposon (mini-Tn5)-driven random transposon mutagenesis combined with the *lux* reporter system. As a proof of concept, we established that disruption of the *sco4804* gene lowers the TopA level, while its overexpression results in enhanced *topA* and gyrase gene transcription. Thus, our approach allowed us to identify a new component of the chromosome supercoiling-related regulatory network in *S. coelicolor*.

## Results

### Application of transposon mutagenesis combined with the *lux* reporter gene system identifies potential regulators of *topA* promoter activity

Our previous studies have shown that *S. coelicolor topA* promoter activity is highly dependent on chromosome supercoiling, but neither negative nor positive protein regulators of the *topA* promoter have been identified [64]. Previously, to detect *topA* activity changes, we used a *lux* reporter plasmid (pFLUX*ptopA*) in which the *topA* promoter controls the transcription of the *luxCDABE* reporter genes [64]. The *luxCDABE* operon encompasses the *luxAB* genes that encode luciferase (a heterodimer of LuxA and LuxB) and the *luxCDE* genes that encode enzymes necessary for luciferase substrate (tetradecanal) biosynthesis [14]. Here, to search for unknown regulators of *topA* promoter activity, we combined *lux* reporter genes and random transposon mutagenesis.

**Fig. 1:**
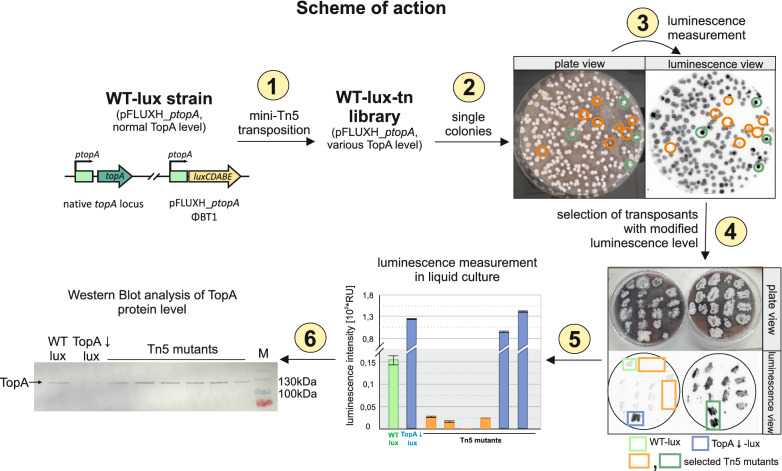
Scheme of random Tn5 transposon mutagenesis in the *S. coelicolor* WT-lux reporter strain. **1** Random transposon mutagenesis of the WT-lux strain (MG03) with a mini-Tn5 transposon. **2** The WT-lux-tn mutant library consisted of approximately 8300 single colonies obtained on MS agar plates. **3** Measurement of the luminescence of WT-lux-tn library single colonies. **4** Selection of colonies with altered light emission compared to the WT-lux paternal strain and TopA-depleted lux strain (MG04, high activity of *topA* promoter). **5** The luminescence of selected colonies from the WT-lux-tn library measured in liquid culture compared with the WT-lux strain and TopA-depleted lux strain (high activity of the *topA* promoter). RU—relative luminescence units. **6** Western blot analysis of TopA protein level in cell lysates of selected colonies from the WT-lux-tn library with anti-TopA polyclonal antibodies. M—molecular mass marker

The *S. coelicolor* WT-lux strain (pFLUX*ptopA* in the wild-type background) was subjected to random transposon mutagenesis (Fig. 1, stage 1), performed using a transposon plasmid (pHL734) containing the mini-Tn-5 transposon [80]. pHL734 harbours a codon-optimized, highly efficient Tn5 transposase (under the control of the *ermE* promoter), which inserts mini-Tn-5 transposons randomly along the chromosome. The conjugation was repeated 4 times to deliver a WT-lux-tn library consisting of at least 8300 single colonies (Fig. 1, stage 2). Since transposition with mini-Tn5 occurs once per genome (as pHL734 cannot replicate in *Streptomyces*), each of the 8300 obtained colonies was assumed to carry a single transposon insertion in the genome. The luminescence of all obtained single colonies was measured during their growth on plates (Fig. 1, stage 3). Next, clones with altered luminescence intensity compared to the paternal WT-lux strain were selected (23 colonies with decreased luminescence and 18 colonies with increased luminescence) and re-streaked on fresh MS plates, and their altered luminescence in comparison to the paternal strain was verified (Fig. 1, stage 4). Subsequently, the clones were cultured in liquid medium, and the luminescence of selected clones was measured again (Fig. 1, stage 5). After the second round of selection, we obtained 12 colonies with significantly lowered or abolished luminescence signals and 2 colonies exhibiting elevated luminescence intensity. The presence of intact *lux* genes in clones with diminished fluorescence was confirmed by PCR (Additional file 1: Fig. S1). Since modification of the *topA* promoter activity detected by changes in *lux* gene activity was also expected to affect the TopA protein level (although earlier we observed that high activity of the *topA* promoter may not lead to high protein level; [64], we next aimed to estimate the TopA level in 14 transposant clones. To this end, the selected clones were cultivated in liquid medium, and the TopA level in the cell lysate was detected using Western blotting with anti-TopA antibodies and compared to the wild-type strain (Fig. 1, stage 6). Lower TopA protein levels (in comparison to those in the wild type strain) were observed in 2 clones, but we did not observe a significant increase in TopA protein level in any of the mutants with elevated luminescence levels. The mutants with altered TopA protein levels were used for further analysis.

In summary, among the transposon mutants, we detected clones with both increased and decreased reporter gene activity. This indicates that the application of transposon libraries in combination with the *lux* reporter genes may be used to identify both positive and negative transcriptional regulators.

### Transposon mutation leads to modified transcription of the *topA* gene but does not affect chromosome supercoiling

**Fig. 2:**
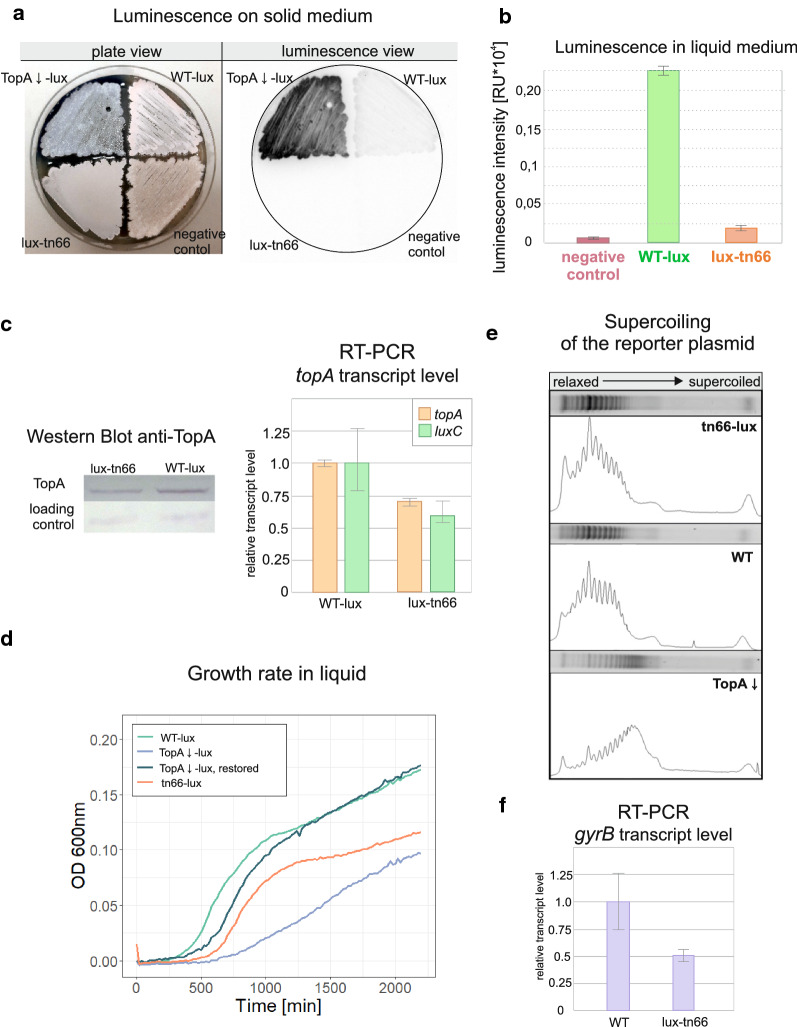
Phenotype of the lux-tn66 transposon strain. **a** Growth and luminescence of the lux-tn66 transposon strain on solid MS medium in comparison to the WT-lux strain (MG03), TopA-depleted lux strain (MG04) and the negative control—wild type strain with empty pFLUXH vector (MG01). Left panel: plate view (after 5 days of growth), right panel: luminescence intensity (after 48 h). **b** Luminescence of mutant reporter strains after 24 h of growth in liquid 79 medium compared to the WT-lux (MG03) and the negative control—wild-type strain with empty pFLUXH vector (MG01). **c** Western blot analysis of TopA protein level (left panel) and the relative transcription of the native *topA* gene, as well as *luxC* reporter gene, in the mutant lux-tn66 strain determined using RT-qPCR analysis performed on 24-h 79 medium cultures, compared to the WT-lux strain (right panel). **d** The growth curves of the lux-tn66 strain (79 medium, Bioscreen C, measurements every 20 min) compared to the WT-lux (MG03) and TopA-depleted lux strain (MG04), as well as to MG04 with restored TopA protein level (after induction with 0.5 µg/ml thiostrepton). **e** Supercoiling density of the reporter plasmids pWHM3Hyg or pWHM3Spec isolated from the transposon mutant lux-tn66 derivative (lux-tn66_RP) strain, the wild-type strain derivative (MS10) and the TopA-depleted (MS11) strain (representative image of two independent experiments). The figure shows topoisomers detected in agarose gel as well as band intensity measurements performed using ImageJ software. **f** The level of *gyrB* transcript in lux-tn66 strain determined using RT-qPCR analysis performed on 24-h 79 medium cultures, compared to the WT-lux strain (MG03)

One of the WT-lux strain transposants, named lux-tn66, emitted very weak luminescence when cultured on solid and in liquid media (Fig. 2a, b). Western blot analyses showed that the level of TopA protein in lux-tn66 was approximately 50% of the protein level in the wild-type strain (Fig. 2c, left), which was also verified by the measurement of *topA* transcript level (Fig. 2c, right). RT-qPCR showed that the *topA* transcript level was approximately 70% of the *topA* transcript level in the wild-type strain. The observed discrepancy between the very low luminescence and moderate lowering of the *topA* transcript level and protein level could suggest either the complex posttranscriptional regulation of the TopA protein level or diminished luciferase activity in the transposon mutant. The RT-qPCR analysis of *luxC* transcript level in the mutant strain compared to the wild type strain confirmed the latter (Fig. 2c, right panel), showing the decrease of *luxC* transcript similar to decrease of *topA* transcript level and much less profound than reduction of luminescence signal. Thus, we infer, that the extensive drop of luminescence in transposon mutant may be caused by transposition-triggered, limited supply of the cofactors for light production. However, the modified level of the *topA* transcript and its protein product confirmed the analysis of the reporter gene activity.

Based on our earlier studies, which showed that lowering the TopA protein level slows the growth of *S. coelicolor* in liquid and solid medium [62], we measured the rate of growth of the lux-tn66 strain. The growth rate analysis in liquid culture showed a slight retardation of transposon strain growth compared to the WT-lux paternal strain, but the growth rate of the transposon strain was still significantly faster than that of the TopA-depleted strain (in which the TopA protein level was approximately 20-fold lower than the wild-type TopA level [62] (Fig. 2d).

Since the severe TopA depletion increases chromosome supercoiling [62], we checked whether decreased TopA protein levels in the transposon lux-tn66 strain caused any changes in global DNA supercoiling. To this end, we determined the level of global DNA supercoiling using modified strains containing the reporter plasmids pWHM3Hyg or pWHM3Spec. We compared the supercoiling of plasmids isolated from modified lux-tn66 (lux-tn66-RP), the wild-type strain derivative (MS10) and the TopA-depleted strain derivative (MS11), which was used as a positive control. DNA supercoiling in transposon mutant lux-tn66-RP was found to be unaffected by decreased TopA protein level (Fig. 2e). Since chromosome supercoiling is maintained by concerted action of gyrase and TopA, we expected that unaltered DNA supercoiling despite the lowered TopA protein level in the lux-tn66 strain may result from changes in gyrase protein level. To test this hypothesis, we determined the activity of the *gyrB* gene encoding one of the gyrase subunits. RT-qPCR analysis of the *gyrB* transcript level in the lux-tn66 transposon mutant showed significantly lower transcription level than wild-type *gyrB* transcription (Fig. 2f). This observation suggests that the lowered gyrase protein level compensated for the undesirable supercoiling changes triggered by the lowered TopA protein level in the lux-tn66 transposon mutant.

Based on our experiments, we infer that the modified level of *topA* gene transcription may either be linked to inactivation of the direct regulator of *topA* gene expression or to the indirect regulation of *topA* involving changes in the gyrase protein level in the mutant strain.

### SCO4804 is a potential candidate for a *topA* promoter activator/regulator

**Fig. 3:**
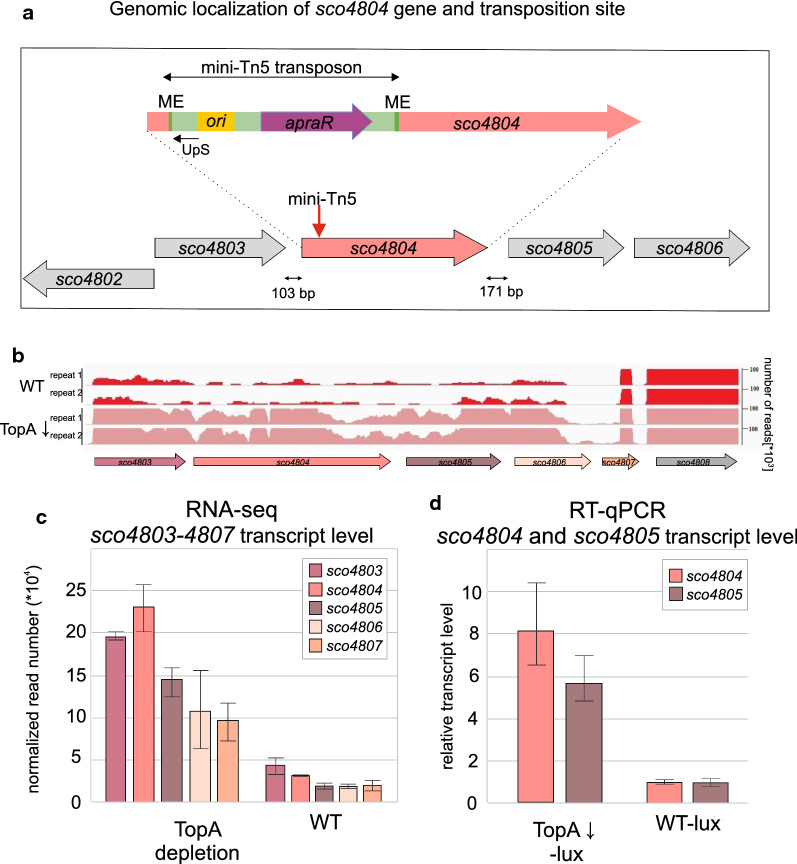
Genomic localization and supercoiling-dependent transcription of *sco4804*. **a** Transposition site in lux-tn66 strain. ME (dark green)—the mosaic end sequence; *ori* (yellow)—origin of replication from pUC vector for DNA replication in *E. coli*; *apraR* (dark violet)—apramycin resistance gene; UpS—primer used for recognition of mini-Tn5 insertion site. The red arrow shows the identified site of the mini-Tn5 insertion. The black arrows at the bottom of the scheme show the distance between neighbouring genes. **b** The transcription profile of *sco4803*-*sco4808* genes in wild type strain (WT) and TopA- depleted strain (PS04, TopA↓) based on RNA-seq experiment data [65], visualized by IGV Viewer. **c** RNA-Seq-based analysis of the expression level of *sco4803*-*sco4806*in the TopA-depleted (PS04) and control wild-type (M145) strains performed for 18-h YEME/TSB cultures, normalized by the upper quartile [65]. The error bars correspond to standard deviations calculated for two independent biological replicates. **d** The relative transcription of *sco4804* and *sco4805* in the TopA-depleted lux (MG04) and WT-lux reporter (MG03) strains calculated using RT-qPCR analysis performed for 24-h cultures in 79 medium

Analysis of the transposon insertion site in the lux-tn66 strain (performed as described by [80] showed that transposition occurred within the *sco4804* gene, 85 bp downstream of its predicted start codon (Fig. 3a). The *sco4804* gene encodes a hypothetical protein, SCO4804, composed of 815 amino acids (predicted molar mass 86.04 kDa), that is rich in glycine and proline residues, and that is conserved in *Streptomyces* species. Structural prediction was performed using Robetta software [55] and indicated the presence of putative alpha-helical structures in the central region of the protein and unstructured regions at both the C- and N-termini. Another analysis performed using PredictProtein [81], also showed three possible DNA-binding regions within the SCO4804 protein structure, which indicates that this protein may act as a transcriptional regulator (Additional file 2: Fig. S2). Comparative analysis in the HOGENOM database [52] showed only a few homologues in other bacterial families (such as Alphaproteobacteria, Bacteroidetes and Cyanobacteria), however, no annotated role was provided for the protein in any species.

Positioning of the *sco4804* gene (103 bp and 171 bp of non-coding regions upstream and downstream of the *sco4804* gene, respectively) suggests that it may not form an operon with adjacent genes (*sco4803* and *sco4805*); however, its genomic location is conserved within the *Streptomyces* genus. SCO4803 and SCO4805 are annotated as hypothetical proteins while SCO4806 as secreted protein. Prediction of SCO4803 and SCO4805 structure [81] showed possible DNA binding domains, but also domains responsible for interactions with other proteins (Additional file 2: Fig. S2). RNA-seq experiments performed previously using *S. coelicolor* wild-type and TopA-depleted strains [65] showed significant, eightfold induction of the *sco4804* gene, as well as adjacent genes, under TopA-depleted conditions (Fig. 3b, c). This result was confirmed for *sco4804* and *sco4805* by RT-qPCR experiments using the WT-lux strain (MG03) and TopA-depleted lux strain (MG04) (Fig. 3d). This strongly suggests that transcription of these genes is supercoiling-dependent, corroborating their potential function in controlling topoisomerase levels.

Markedly, RT-qPCR analysis revealed significant decrease of *sco4805* transcription in the lux-tn66 transposon strain suggesting a polar effect of transposition within *sco4804* gene. This indicates that either product of one of those two genes is a *topA* promoter regulator or both their products act in cooperation to fulfill this role.

### SCO4804 overproduction increases *topA* promoter activity

Since *sco4804* transcription was shown to be induced by TopA protein depletion and disruption of the *sco4804* gene lowered TopA protein level, we predicted that SCO4804 acts as a positive regulator of *topA* transcription. To confirm this hypothesis, we constructed a strain overexpressing the *sco4804* gene in the WT-lux strain background.

**Fig. 4:**
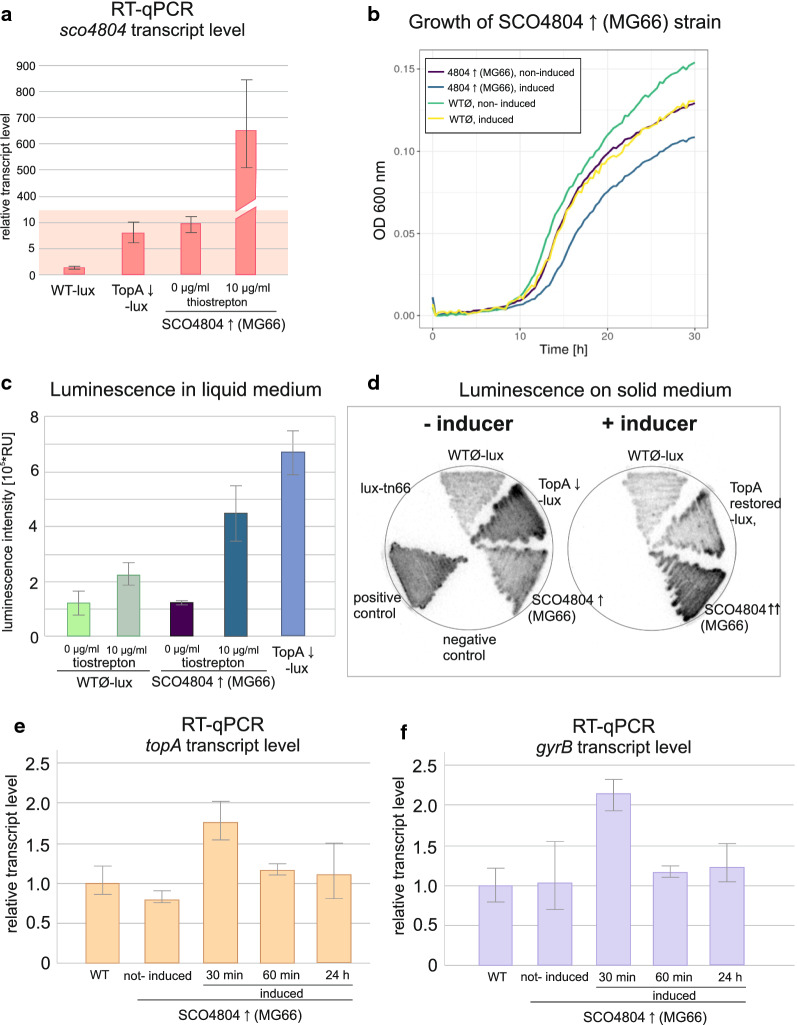
Phenotype of the SCO4804 overproducing strain. **a** RT-qPCR analysis of the *sco4804* transcript level in the MG66 strain (non-induced and induced with 10 µg/ml thiostrepton) compared to the WT-lux (MG03) and TopA-depleted lux (MG04) strains performed for 24 h cultures grown in 79 medium. **b** The growth curves of the non-induced MG66 strain and MG66 induced with 10 µg/ml thiostrepton (79 medium, Bioscreen C, measurements every 20 min) compared to the WTØ strain (M145_pIJ6902). **c** Measurement of luminescence of the MG66 strain in the absence or in the presence of the *sco4804* inducer (0 and 10 μg/ml of thiostrepton) indicating the changes of *topA* promoter activity, performed in liquid 79 medium after 24 h of growth and compared to the control strain (WTØ-lux, MG03_pIJ6902) that contains empty pIJ6902 plasmid (and TopA- depleted lux (MGO4) strains. **d** Luminescence of MG66 indicating the activity of the *topA* promoter controlling the *lux* reporter genes after 48 h of growth on solid MS agar plates, without and with the inducer (10 µg/ml thiostrepton), as compared to the control strain (WTØ-lux, MG03_pIJ6902) that contains empty pIJ6902 plasmid (and TopA-depleted lux (MGO4) strain, negative control (the wild-type strain with empty pFLUXH vector (MG01)) and positive control (the wild type strain with pFLUXH_permE (MG02)). **e** RT-qPCR analysis of the relative transcription of the *topA* gene in the SCO4804 overproducing strain (MG66) cultured in 79 medium for 24 h and induced with 10 µg/ml thiostrepton for 30 min, 60 min or cultured for 24 h in the presence of the inducer. The data were compared to the non-induced control and WT-lux strain (MG03) grown for 24 h in 79 medium. **f** RT-qPCR analysis of the relative transcription of the *gyrB* gene in the SCO4804-overproducing strain (MG66) induced after 24 h of growth with 10 µg/ml thiostrepton for 30 min and 60 min and/or cultured for 24 h in the presence of the inducer. The data were compared to the non-induced control and WT-lux strain (MG03) grown for 24 h in 79 medium

In the obtained strain (MG66), *sco4804* (as a second gene copy in the integrative vector pIJ6902) was controlled by a thiostrepton-inducible *tipA* promoter. Overexpression of *sco4804* in MG66 cells was confirmed by RT-qPCR, revealing significantly elevated *sco4804* transcript levels in comparison to the WT-lux strain background (Fig. 4a). However, we also observed significant induction of *sco4805* gene transcription in response to *sco4804* overexpression (Additional file 3: Fig. S3). Th observation that SCO4804 controls *sco4805* transcription reinforces the notion that both proteins act in cooperation.

Having confirmed the induction of *sco4804* in the MG66 strain, we set out to analyse the influence of SCO4804 on growth and *topA* promoter activity, as well as DNA supercoiling. Induction of *sco4804* led to slight retardation of growth compared to the control WTØ strain (with pIJ6902 empty vector) (Fig. 4b). The *topA* promoter activity, measured using the *lux* reporter genes, was significantly increased by *sco4804* induction either in 24-h liquid or 48-h plate cultures of the MG66 strain cultured in the presence of inducer (10 µg/ml thiostrepton) (Fig. 4c, d). We excluded the effect of thiostrepton on the activity of *topA* promoter and *luxCDABE* operon in induced *sco4804* overexpressing strain by checking the luminescence as well as *topA* transcription in response to induction in the WTØ-lux strain (Fig. 4c, d and Additional file 4: Fig. S4). Thus, the obtained results indicated that overexpression of *sco4804* caused significant activation of the *topA* promoter.

Next, to confirm that *sco4804* is a positive regulator of the *topA* promoter, we performed RT-qPCR analysis of *topA* transcript level in the MG66 strain with induced *sco4804* overexpression. The results showed that increased *topA* transcript level was observed immediately after induction of *sco4804* (30 min after the addition of thiostrepton to the medium), but after 60 min of incubation in the presence of inducer, *topA* transcript level decreased to the wild-type level (Fig. 4e). The discrepancy between long-term elevated *lux* activity after *sco4804* induction and *topA* transcript elevation only during a very short period of time after induction suggests other post-transcriptional regulation modifications of *topA* transcript level, reinforcing our previous experiments [65]. Interestingly, the level of *gyrB* transcript exhibited similar changes, with an increase 30 min after *sco4804* induction and a decrease 60 min after induction (Fig. 4f). This indicates that the balance between gyrase and TopA activities was established and that the native supercoiling level could be restored. Indeed, the analysis of the reporter plasmid supercoiling showed no changes in the MG66 strain upon *sco4804* induction (Additional file 5: Fig. S5).

To check whether SCO4804 is a direct regulator of *topA* promoter activity, we tested its binding to the *topA* promoter in vitro. To this end, we purified the 6His-SCO4804 protein using the *E. coli* BL21 (DE3) groEL-groES strain [24, 51] (Supplementary info, Additional file 6: Fig. S6A) and performed an electrophoretic mobility shift assay (EMSA) using a 458 bp DNA fragment encompassing the *topA* promoter and 632 bp promoter of the *sco4697* gene, as well as 654 bp of a part of the *sco3928* gene as the negative controls. While 6His-SCO4804 bound all tested DNA fragments at a concentration of 1 µM, it was non-specific towards the *topA* promoter (Additional file 6: Fig. S6B). Moreover, the addition of poly(dIdC) competitor DNA eliminated all non-specific interactions. A further pull-down assay and topoisomerase activity tests [63] in the presence of 6His-SCO4804 excluded the possibility of a direct interaction between SCO4804 and TopA (Additional file 7: Figs. S7 and Additional file 8: S8). These experiments suggest that SCO4804 influences *topA* promoter activity in an indirect manner.

## Discussion

Our approach combining *lux* reporter genes and a random transposon library allowed us to perform high-throughput screening for potential regulatory proteins that control TopA protein level in *S. coelicolor*. Genome-wide transposition, as a powerful genetic tool, is widely used for systematic genetic studies of different bacterial species, including the construction of random insertion *Streptomyces* mutants with IS6100, Tn4560, IS493, Tn5, and Himar1 transposons [4, 43, 70, 72, 75]. The Tn5 minitransposon together with the codon-optimized Tn5 transposase displays high efficiency, less codon bias and lower host specificity than other transposases [80]. In *Streptomyces*, random mutagenesis has previously been used to find repressors or activators of genes of interest, the products of which are easy to monitor within the cell, such as antibiotic or pigment production. This technique has been successfully applied for the identification of actinorhodin and landomycin E negative regulators [13, 29]. Moreover, transposon mutagenesis combined with a reporter system based on an antibiotic resistance cassette was previously applied to search for repressors for daptomycin production in *S. roseosporus* [40]. However, this approach, based on antibiotic resistance genes, limited the screening to negative transcriptional regulators. The advantage of our, in comparison to the abovementioned approaches, is its suitability for high-throughput searches of both negative and positive regulators in *Streptomyces* transposon libraries. The strategy described here may be used for challenging identification of global regulators that control secondary metabolite synthesis pathways. Thanks to the application of *lux* reporter genes fused to promoter(s) of either regulatory or biosynthetic gene(s) from secondary metabolite cluster, identification of its global regulator(s) may not rely on detection of the product of interest but on easily performed luminescence measurements. Moreover, the changes in gene of interest expression can be readily monitored in both liquid and solid cultures over time and in different environmental conditions, which also enables the identification of regulators active only in particular environmental conditions. However, it must be considered that this method is limited to non-essential regulators. The other disadvantage of using *lux* reporter genes is the formation of artefacts due to metabolic influences on luciferase activity (affected by changes in oxygen, ATP, Mg ions levels), but this can be overcome by using a second reporter system, for example, based on *gfp, gusA, lacZ* expression [2, 9, 35, 47], which, although not that convenient as *lux* reporter in terms of measurements, are less dependent on metabolic state. Nevertheless, because of the compatibility of all genetic elements, we believe that the strategy tested here for the identification of regulators may be widely used in *Streptomyces*.

By applying our screening approach, we expected to find any proteins that influence TopA protein level with either transcriptional or translational/post-translational modes of action. It was shown earlier that the *topA* promoter was activated by increased chromosome supercoiling and was inhibited due to chromosome relaxation after novobiocin treatment [64]. In addition to supercoiling sensitivity, no other factor controlling promoter activity has been identified to date,thus, the identification of either a *topA* activator or repressor was of interest. We found that SCO4804 (possibly in cooperation with SCO4805) acts as a *topA* transcriptional activator, since its elimination decreased *topA* promoter activity and protein level, while induction of *sco4804* (and related to this *sco4805* overexpression) resulted in higher activity of the *topA* promoter. Notably, our previous studies showed that elevated *topA* transcript level does not correspond with elevated TopA protein level, as well as or with significant changes in DNA supercoiling levels [64]. However, the fact that the *topA* transcript level increased and subsequently diminished shortly after SCO4804 induction suggests that other mechanisms of maintaining TopA protein level are also activated. We previously suggested that the *topA* transcript and TopA protein levels are controlled by multiple regulatory strategies that act concertedly to preserve constant supercoiling level in *Streptomyces* [64, 66]. Additionally, we observed that *sco4804* induction influences not only TopA but also gyrase genes expression, indicating that the newly identified protein may be a component of the chromosome supercoiling maintenance system. The fact that transcription of *sco4804* is activated in response to increased negative supercoiling corroborates its potential function as the regulator of topoisomerase activity. Moreover, the supercoiling activated transcription of the neighbouring genes (*sco4803* and *sco4805*) suggest that their products may act in a cooperation with SCO4804.

## Conclusions

To summarize, our screening approach is optimized for *Streptomyces* and allows the identification of both positive and negative regulators that control the expression of genes of interest by either direct or indirect mechanisms. As proven by our concept, the protein SCO4804 was found to be a component of a complex regulatory network involved in *S. coelicolor* chromosome supercoiling maintenance. Since the production of secondary metabolites is regulated by chromosomal topology, understanding complex transcriptional regulation in *Streptomyces* is crucial for the industrial application of these bacteria.

## Methods

### Bacterial strains, plasmids, and growth conditions

Basic DNA manipulation procedures were performed according to standard protocols [60]. Unless otherwise stated, all enzymes and isolation kits were obtained from Thermo Fisher Scientific (Waltham, MA) and NEB (Ipswitch, MA). Bacterial media and antibiotics were purchased from Difco Laboratories (Detroit, MI) and Carl Roth (Karlsruhe, Germany), respectively. The *S. coelicolor* growth conditions and antibiotic concentrations, as well as the conjugation procedure, followed the general protocols described by [34]. For induction, thiostrepton at concentrations of 0.5–10 µg/ml was added. Growth curves of the *S. coelicolor* strains were determined using the Bioscreen C device (Oy Growth Curves Ab Ltd., Helsinki, Finland). Cultures were grown in triplicate in 79 medium [54] (300 µl/well), inoculated with 0.01 U/ml spores (1 U is defined as the volume of spore stock solution diluted up to 1 ml with OD_600nm_ = 1). The *S. coelicolor* and *E. coli* strains used in this study are shown in Table 1. The plasmids used in this study are shown in Additional file 9: Table S1.

**Table 1:** Strains used in this study

Name	Genotype	Source or reference
*S. coelicolor*		
M145 (WT)	SCP1-SCP2-	[3]
PS04 (TopA↓)	M145 Δ*topA*::scar *attB* ΦC31::pIJ6902*topA*	[62]
MG03 (WT-lux)	M145 attBΦBT1::pFLUXH*ptopA*	[64]
MG04 (TopA↓-lux)	M145 Δ*topA*::scar attBΦC31::pIJ6902*topA* attBΦBT1::pFLUXH*ptopA*	[64]
MG02	M145 attBΦBT1::pFLUXH_*permE*	[64]
MG01	M145 attBΦBT1::pFLUXH	[64]
lux-tn66	MG01 *tn5*::*sco4804*	This study
lux-tn66RP	lux-tn66 pWHM3Spec	This study
MS10	M145 pWHM3Hyg	[64]
MS11	M145 Δ*topA*::scar attBΦC31::pIJ6902*topA* pWHM3Hyg	[62]
MG66	MG01 attBΦC31::pIJ6902_*sco4804*	This study
MG66_RP	MG66 pWHM3Hyg	This study
M145_pIJ6902 (WTØ)	M145 attBΦC31::pIJ6902	This study
MG03_pIJ6902 (WTØ-lux)	MG03 attBΦC31::pIJ6902	This study
*E. coli*		
DH5α	F–endA1 glnV44 thi-1 *recA1 relA1 gyrA96 deoR nupG* purB20 φ80dlacZΔM15 Δ(*lacZ*YA-argF)U169, *hsdR*17(rK–mK +), λ–	Lab stock
ET12567 pUZ8002	*dam*-13::Tn*9 dcm cat tet hsd zjj*-*201*::Tn*10 tra neo* RP4	[34]
BW25113/pIJ790	K12 derivative; Δ*araBAD* Δ*rhaBAD* λ-Red (*gam bet exo*) *cat araC rep101*(Ts)	Gust et al. [26]
BL21 (DE3) groEL-groES strain	F–*ompT gal dcm lon hsdSB*(rB–mB–) λ(DE3 [*lacI lacU*V5-T7p07 ind1 sam7 nin5]) [*malB* +]K-12(λS) pGroESL	[24]

### Transposon mutagenesis

To perform random transposon mutagenesis in *S. coelicolor* MG03, we used the pHL734 vector. Mutagenesis was performed according to a procedure described earlier [80]. Briefly, *E. coli* ET12567 pUZ8002 harbouring the pHL734 plasmid was grown to OD_600nm_ = 0.5 and conjugated with 5 U of *S. coelicolor* MG03 (WT-lux) spores. The conjugated cell mixture was diluted (10^–4^–10^–6^) and plated on MS agar supplemented with 10 mM MgCl_2_ and 60 mM CaCl_2_ to obtain single colonies. After 17 h of growth at 30 °C, the plates were overlaid with 20 µl per plate of each antibiotic: nalidixic acid (25 mg/ml), apramycin (50 mg/ml) and hygromycin B (50 mg/ml). The obtained colonies were tested for luminescence intensity (see below), re-streaked on fresh MS agar plates supplemented with apramycin and hygromycin and used to establish liquid cultures. The positions of mini-Tn5 insertions in the *S. coelicolor* MG03 chromosome were identified using the rescue plasmid method. First, chromosomal DNA was isolated from a 24-h culture in 79 medium of *S. coelicolor* transposon strains. Subsequently, 2 µg of chromosomal DNA was digested with the ApaI restriction enzyme (at 37 °C overnight, 50 µl total reaction volume), and then DNA was purified using a CleanUp kit (A&A Biotechnology, Gdynia, Poland) and eluted with 15 µl of ultrapure water. Then, 100–200 ng of the ApaI-digested DNA was re-ligated (at 4 °C overnight, 20 µl total volume of the reaction) using 1 µl of T4 DNA ligase (NEB) to allow formation of a mini–*E*. *coli* replicative plasmid. Electrocompetent *E. coli* DH5α cells were transformed with the ligation products (using half of the reaction volume) and selected on apramycin LB agar plates. Plasmid DNA was isolated from single *E. coli* colonies using a Plasmid Screening Kit (Syngen Biotech, Wrocław, Poland) according to the manufacturer’s instructions. The isolated plasmids were digested with the ApaI restriction enzyme and analysed using gel electrophoresis, and plasmids exhibiting different digestion patterns were picked for subsequent DNA sequencing. Sequencing (Sigma-Aldrich, Saint Louis, MO) using UpS oligonucleotides identified the sites of mini-Tn5 insertion.

### Strain construction

For inducible overexpression of the *sco4804* gene, the pIJ6902_*sco4804* plasmid was constructed. A DNA fragment encompassing the *sco4804* gene with flanking EcoRI and NdeI restriction sites was synthesized and cloned into the pUC57 mini plasmid, yielding pUCmini_4804 (GenScript Biotech Corporation, New Jersey, US). The pIJ6902_*sco4804* plasmid was obtained by restriction cloning of the *sco4804* insert into the pIJ6902 plasmid using NdeI and EcoRI sites. The construct was then conjugated from *E. coli* ET12567 pUZ8002 into *S. coelicolor* MG03 (WT-lux strain), apramycin-resistant exconjugants were selected, and the plasmid presence in the obtained strain MG66 was confirmed by PCR using M13pUCr and sco4804_rv oligonucleotides.

To analyse DNA supercoiling in the *S. coelicolor* transposon mutant lux-tn66 strain, we modified the pWHM3Hyg reporter plasmid [62] by substituting the hygromycin resistance cassette with the spectinomycin resistance gene using the Redirect system [26], oligonucleotides spect_fwd_2 and spect_rv and plasmid pIJ778 as a template, yielding pWHM3Spec. Next, we introduced the pWHM3Spec plasmid into the lux-tn66 transposon mutant strain via conjugation with *E. coli* ET12567 pUZ8002 [34]. The MG66_RP strain, which was also used for analysis of DNA supercoiling, was obtained by conjugation of the pIJ6902_*sco4804* plasmid into the MS10 strain (WT harbouring the pWHM3Hyg reporter plasmid).

As a control for the analysis of the MG66 strain induced with thiostrepton, we also constructed the M145_pIJ6902 and MG03_pIJ6902 strains, in which the empty plasmid pIJ6902 was introduced via conjugation into the M145 and MG03 *S. coelicolor* strains, respectively.

### Reporter gene activity assays

To measure luciferase activity in liquid culture, strains containing the *luxCDAEB* operon (under the control of the *topA* promoter or under the control of the *erm* promoter) in the pFLUXH ΦBT1 integrating vector were grown in liquid 79 medium for 24 h at 30 °C (in three biological replicates for each strain). Subsequently, the mycelium was collected by centrifugation, wet weight was determined, and mycelium was resuspended in 300 µl of 79 medium. Measurement of the luciferase activity was performed in triplicate directly from the mycelium suspension for each biological sample in a 100 µl volume in 96-well microplates (Perkin Elmer, Waltham, MA) using the Infinite PRO Multimode Plate Reader (Tecan, Männedorf, Switzerland). The luminescence intensity was normalized against wet weight (units/100 mg of mycelium). Luminescence visualization on solid medium was performed on MS agar plates after 48 h of growth at 30 °C, and luminescence detection was performed using a ChemiDocXRS + device (Bio-Rad, Hercules, CA).

### RNA isolation and RT-qPCR

For gene expression analysis, RNA was isolated from *S. coelicolor* cultures grown for 18 h (unless otherwise stated) in liquid 79 medium. Before harvesting, a 1/10 culture volume of 95% EtOH saturated with phenol was added at a 5% final concentration to stabilize cellular RNA [57], and then mycelium was harvested by centrifugation and frozen in liquid nitrogen. Next, total RNA was isolated using Tri-Reagent (Sigma-Aldrich) according to the manufacturer’s protocol. The RNA solution was transferred to a Total RNA Mini column (A&A Biotechnology) and processed according to the manufacturer’s instructions. The RNA samples were digested with Turbo DNase I (Invitrogen, Carlsbad, CA) to remove traces of chromosomal DNA and then purified and concentrated using Clean-Up RNA Concentration (A&A Biotechnology). Five hundred micrograms of RNA was used for cDNA synthesis with the Maxima First Strand cDNA Synthesis Kit (Thermo Fisher Scientific). cDNA samples were diluted 5 times and used as templates for quantitative PCR (qPCR, each reaction performed in triplicate) using PowerUp SYBR Green Master Mix (Thermo Fisher Scientific). The level of the *topA*, *gyrB, sco4804*, *sco4805* and *luxC* transcript was quantified using *hrdB* as a reference gene (ΔΔCT method) (StepOnePlus Real-Time PCR system; Applied Biosystems, Foster City, CA) (Additional file 9: Table S2). Isolated RNA was tested for DNA contamination by qPCR with oligonucleotides complementary to the *S. coelicolor hrdB* gene. The difference > 5 Ct after 30 PCR cycles between the RNA sample and the corresponding cDNA sample as a template showed that the RNA samples were DNA-free.

### DNA supercoiling assay

Global DNA supercoiling in *S. coelicolor* strains was quantified using the pWHM3Hyg reporter plasmid [62] or its modified version, pWHM3Spec (Table S1). The plasmids were isolated using alkaline lysis and column purification based on a modified version of the manufacturer’s (Plasmid Screening Kit, Syngen) procedure. After 48 h of growth in liquid 79 medium supplemented with hygromycin or spectinomycin, *S*. *coelicolor* mycelium was collected by centrifugation, resuspended in PZ buffer containing 25 mg/ml lysozyme and incubated at 30 °C for 5 min. The subsequent steps followed the manufacturer’s protocol. The isolated reporter plasmids were resolved in 0.8% agarose in Tris–acetate-EDTA (TAE) buffer (40 mM Tris, 20 mM acetic acid, and 1 mM EDTA, pH 8.3) in the presence of 4.6 µM chloroquine at a voltage of 20 V. To visualize topoisomers, the gel was stained with ethidium bromide for 30 min at room temperature. The experiment was repeated twice. The topoisomer distribution was analysed using ImageJ software.

### TopA level quantification using Western blotting

For TopA level quantification, *S. coelicolor* 5 ml liquid cultures in 79 medium were cultivated for 24 h. Next, the cell pellet was collected by centrifugation, resuspended in phosphate-buffered saline (PBS), sonicated and centrifuged. The cell lysates (5 µg of total protein) were separated by 10% SDS-PAGE according to standard procedures [37]. After electrophoresis, the resolved proteins were stained overnight with PageBlue Protein Staining Solution (Thermo Fisher Scientific) or transferred to a nitrocellulose membrane and blocked with 2% milk in TBST (20 mM Tris, 150 mM NaCl, 0.1% Tween 20, pH 7.4–7.6). The blots were subsequently incubated with rabbit polyclonal TopA antiserum (1:10,000 in TBST,1-h incubation; [62] and visualized using alkaline phosphatase-conjugated goat anti-rabbit antibodies (1:5000) (Sigma-Aldrich). The band intensities were analysed using ImageJ software, comparing the TopA band intensity of particular mutants to the wild-type reference.

### Structure prediction and homologue analysis

Protein structure predictions were performed using the Robetta Web Server and “TrRefineRosetta” modelling method [55, 61], as well as using PredictProtein [81]. Homologue searching was performed using HOGENOM [52].

## Supplementary Information

**Additional file 1: Fig. S1 PCR confirming the presence of the pFLUXH integrated vector in clones from the WT-lux-tn library.** PCR was performed on *S. coelicolor* colonies using topA_p1_fw and luxC_rv oligonucleotides. The amplicon (499 bp) is marked with a black arrow. M—DNA molecular mass marker.

**Additional file 2: Fig. S2 Structural analysis of the SCO4804, SCO4803 and SCO4805 proteins using PredictProtein software**. The image shows the localization of the predicted secondary structures, DNA-binding domains (RI—reliability index reflecting the strength of a prediction, high value means high confidence for binding) and the predicted disordered regions. In the case of SCO4803 and SCO4805 software predicted also some protein binding regions, which are also included to the scheme.

**Additional file 3: Fig. S3** RT-qPCR analysis of the relative transcription of the *sco4805* gene in the lux-tn66 transposon mutant as well as in *sco4804* overexpressing strain (MG66) cultured in 79 medium for 24 h and induced with 10 µg/ml thiostrepton, compared to the non-induced control and WT-lux strain (MG03) grown for 24 h in 79 medium.

**Additional file 4: Fig. S4** RT-qPCR analysis of the relative transcription of the *topA* gene in the control strain (WTØ-lux) containing empty pIJ6902 plasmid (MG03_pIJ6902) overproducing strain cultured in 79 medium for 24 h and induced with 10 µg/ml thiostrepton. The data were compared to the non-induced control grown for 24 h in 79 medium.

**Additional file 5: Fig. S5 DNA supercoiling in the *sco4804* overexpressing strain.** DNA supercoiling density of the reporter plasmid pWHM3Hyg isolated from the *sco4804* overexpressing MG66 strain derivative (MG66_RP), induced with 10 µg/ml thiostrepton for 45 min or cultured for 24 h in the presence of inducer compared to the non-induced control, the wild-type strain derivative (MS10) and the TopA-depleted strain derivative (MS11) (representative images of two independent replicates are shown). The figure shows topoisomers detected in agarose gel as well as band intensity measurements performed using ImageJ software.

**Additional file 6: Fig. S6 Purification of 6His-SCO4804 recombinant protein and DNA binding analysis. A**. SDS-PAGE analysis of the collected fractions obtained during 6His-SCO4804 purification from *E. coli* BL21 (DE3) groEL-groES. M—Molecular mass marker, 1—non-induced *E. coli* cell extract, 2—induced *E. coli* cell extract in sarcosyl buffer, 3—induced *E. coli* cell extract in binding buffer, 4—induced *E. coli* cell extract in binding buffer, soluble fraction, 5—flow-through, 6—proteins bound to Ni–NTA agarose, 7—wash with binding buffer with 40 mM imidazole, 8—proteins eluted by 200 mM imidazole, 9—the resin after elution. **B**. Electrophoretic mobility shift assay (EMSA) performed with 30 ng of 461 bp dsDNA fragment of the *topA* promoter and two negative controls as follows: negative control 1, a 415 bp DNA fragment encompassing the non-coding region between *sco4696* and *sco4697* genes, and negative control 2, a 654 bp fragment of the *sco3928* gene. Binding was performed in PBS containing 5 mg/ml BSA, 5% (v/v) glycerol and, optionally, 2 ng/µl poly(dI-dC). The samples were resolved on a 5% polyacrylamide gel run at 4 °C in 0.25 × Tris–borate-EDTA (TBE) buffer (22.5 mM Tris, 22.5 mM boric acid, 0.5 mM EDTA) at 100 V for 3–4 h. The bands were visualized with ethidium bromide solution that was incubated for 30 min at room temperature and with a ChemiDoc XRS + system (Bio-Rad).

**Additional file 7: Fig. S7 Gel electrophoresis demonstrating TopA activity in the presence of 6His-SCO4804 recombinant protein.** The assay was performed using 120 ng of TopA and 100 ng of pUC19 plasmid and increasing concentrations of 6His-SCO4804 recombinant protein. The reaction was incubated at 37 °C for 15 min and subsequently stopped by the addition of 2 μl of 0.5 M EDTA. The samples were subsequently resolved on a 0.8% agarose gel in TAE buffer for 14–16 h at low voltage (2 V/cm).

**Additional file 8: Fig. S8 Pull-down experiment using 6His-SCO4804 recombinant protein bound to Ni–NTA agarose resin**. The resin was subsequently incubated with lysate of the TopA-induced PS04 strain (TopA + lysate). The negative controls served as lysates of the TopA-depleted PS04 (TopA-lysate) strain loaded on 6His-SCO4804 – Ni–NTA agarose and Ni–NTA resin lacking immobilized 6His-SCO4804 recombinant protein. The incubation was performed in TN buffer with 40 mM imidazole. Elution of specifically bound protein was performed using 200 mM imidazole in TN buffer. The samples for the Western blot analysis were prepared using 15 µl of lysate fractions and unbound fraction, 10 µl of eluted proteins and 5 µl of resin as a bound fraction. Samples were resolved using SDS-PAGE and visualized using Western blot and anti-TopA polyclonal antibodies as well as 6xHis tag monoclonal antibody (MA1-135, Thermo Fisher Scientific) to confirm efficient 6His-SCO4804 binding to the resin and its subsequent elution.

**Additional file 9.** Detailed informations on heterological overexpression of 6His-SCO4804, in vitro experiments, plasmids and oligonucleotides used in the study.

## Data Availability

The datasets used and analysed during the current study are available from the corresponding author on reasonable request.

## Abbreviations

WT: Wild type
RNAP: RNA polymerase
TF: Transcription factor
TAE: Tris: acetate-EDTA buffer
TBE: Tris–borate-EDTA buffer
MS: Mannitol soya flour agar
PBS: Phosphate-buffered saline
TBST: Tris-buffered saline with 0.1% Tween 20 detergent
