# Supplementary figures and images for: Combining transposon mutagenesis and reporter genes to identify novel regulators of the topA promoter in Streptomyces
Source: Microb Cell Fact. 2021 May 13;20:99. doi: 10.1186/s12934-021-01590-7 (PMC8120823; doi:10.1186/s12934-021-01590-7)

# Fig. S1

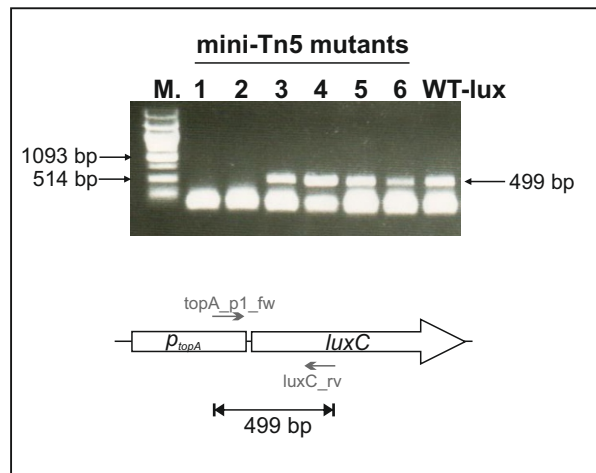

Supplement: Supplementary file 1 — Additional file 1: Fig. S1 PCR confirming the presence of the pFLUXH integrated vector in clones from the WT-lux-tn library. PCR was performed on S. coelicolor colonies using topA_p1_fw and luxC_rv oligonucleotides. The amplicon (499 bp) is marked with a black arrow. M—DNA molecular mass marker. [file 12934_2021_1590_MOESM1_ESM.pdf]

Fig. S2

A

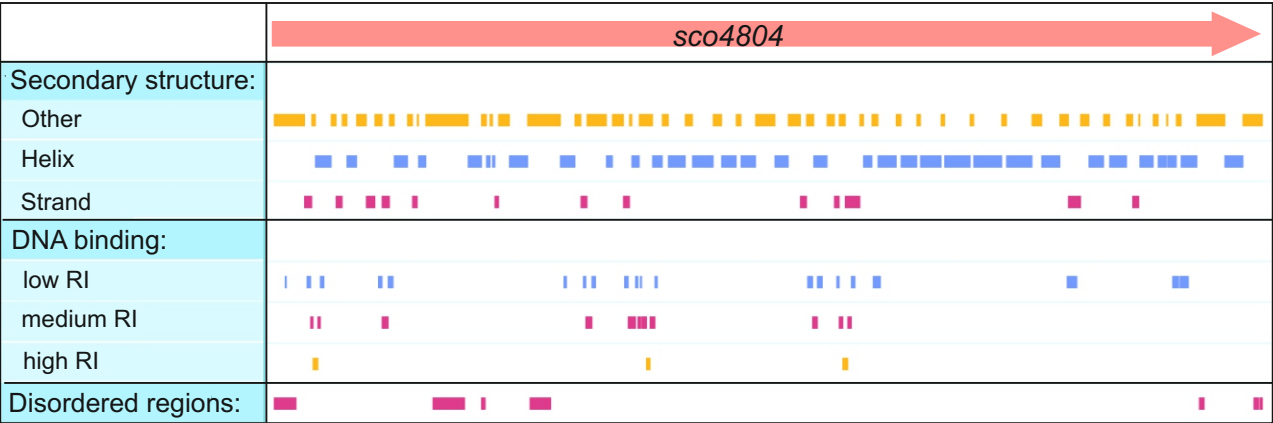

B

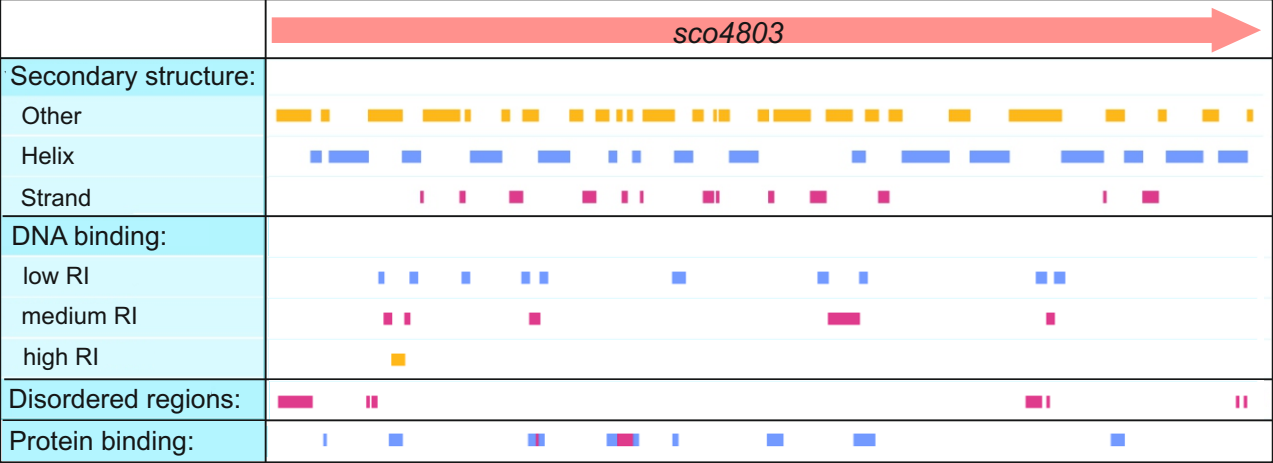

C

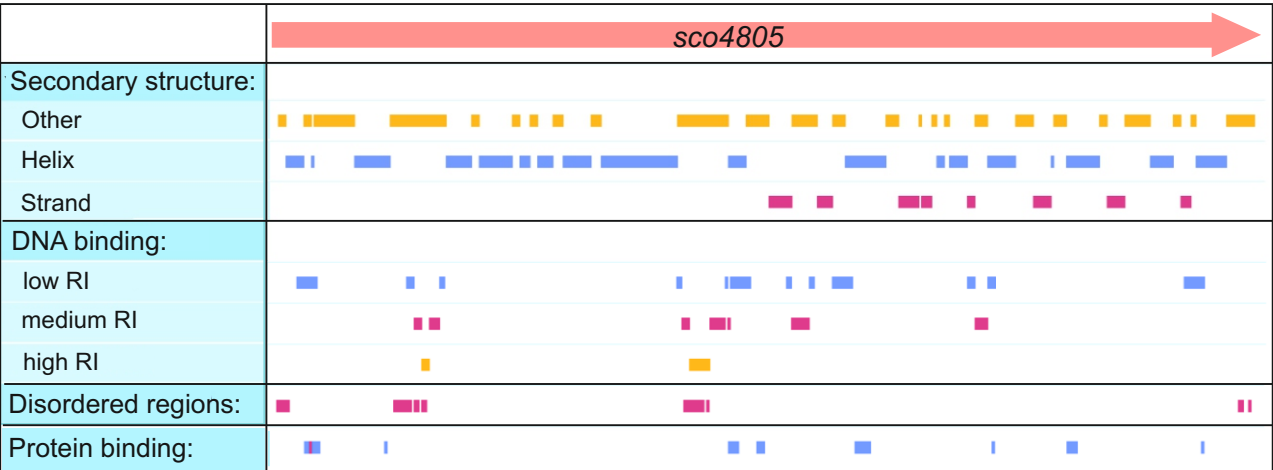

Supplement: Supplementary file 2 — Additional file 2: Fig. S2 Structural analysis of the SCO4804, SCO4803 and SCO4805 proteins using PredictProtein software. The image shows the localization of the predicted secondary structures, DNA-binding domains (RI—reliability index reflecting the strength of a prediction, high value means high confidence for binding) and the predicted disordered regions. In the case of SCO4803 and SCO4805 software predicted also some protein binding regions, which are also included to the scheme. [file 12934_2021_1590_MOESM2_ESM.pdf]

Fig. S3

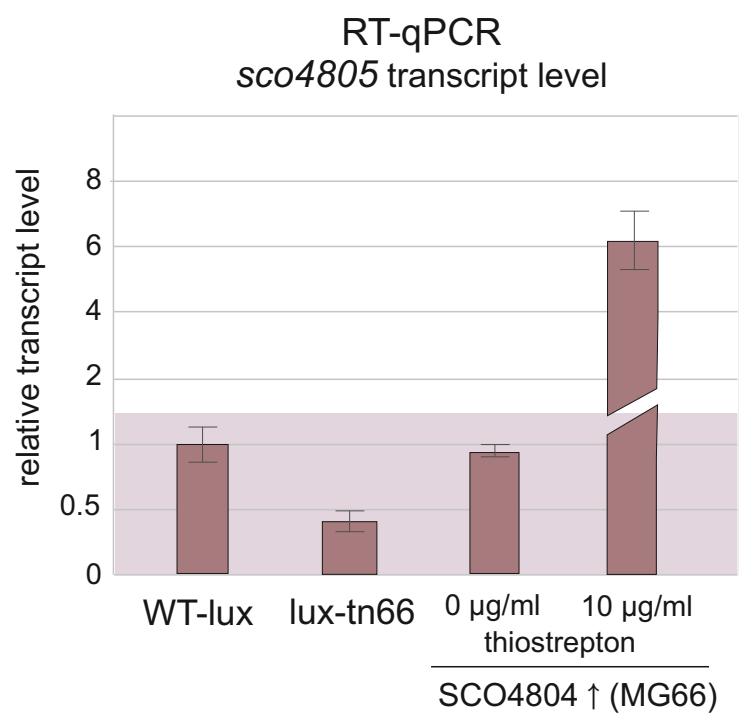

Supplement: Supplementary file 3 — Additional file 3: Fig. S3 RT-qPCR analysis of the relative transcription of the sco4805 gene in the lux-tn66 transposon mutant as well as in sco4804 overexpressing strain (MG66) cultured in 79 medium for 24 h and induced with 10 µg/ml thiostrepton, compared to the non-induced control and WT-lux strain (MG03) grown for 24 h in 79 medium. [file 12934_2021_1590_MOESM3_ESM.pdf]

Fig. S4

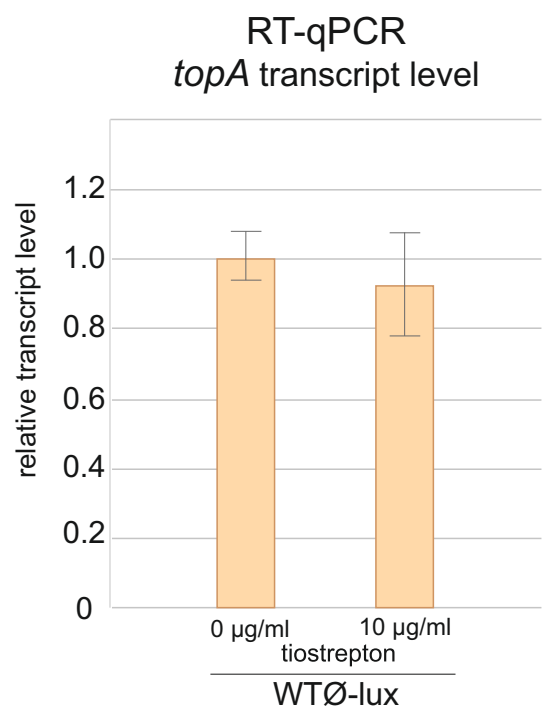

Supplement: Supplementary file 4 — Additional file 4: Fig. S4 RT-qPCR analysis of the relative transcription of the topA gene in the control strain (WTØ-lux) containing empty pIJ6902 plasmid (MG03_pIJ6902) overproducing strain cultured in 79 medium for 24 h and induced with 10 µg/ml thiostrepton. The data were compared to the non-induced control grown for 24 h in 79 medium. [file 12934_2021_1590_MOESM4_ESM.pdf]

**Fig. S5**

DNA supercoiling of the reporter plasmid  
in the MG66 (SCO4804  $\uparrow$ ) strain

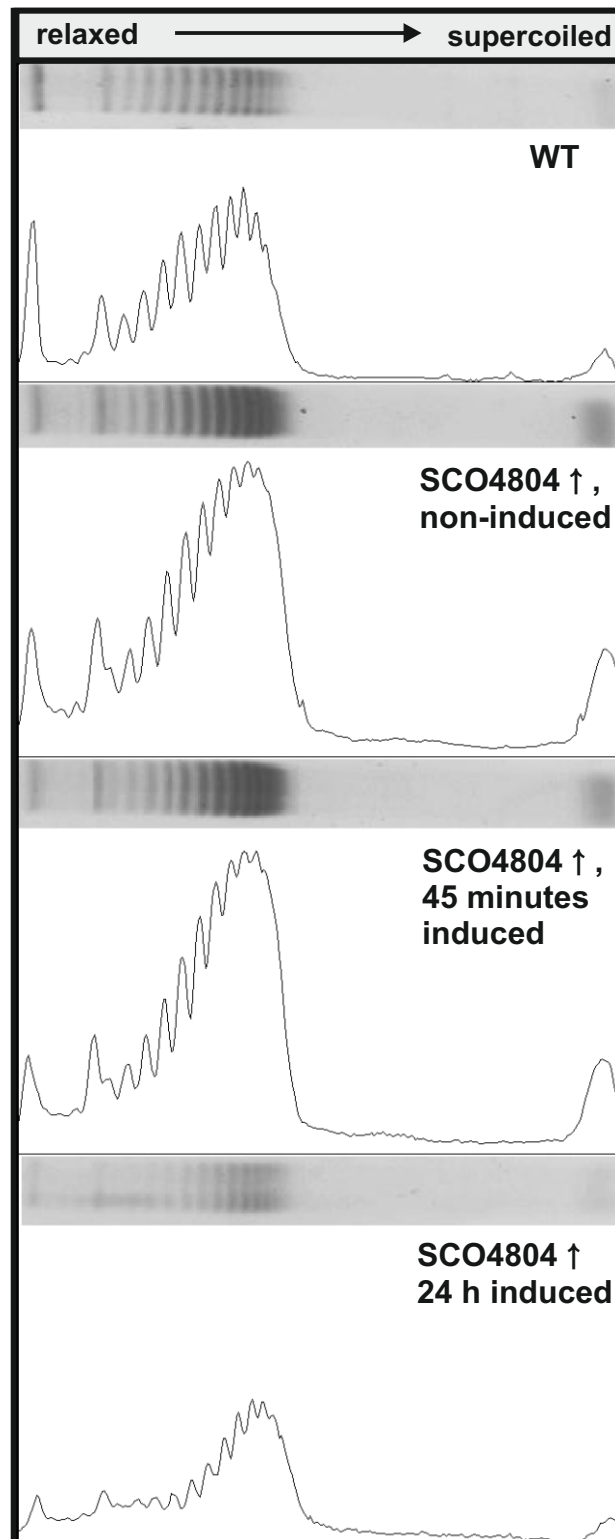

Supplement: Supplementary file 5 — Additional file 5: Fig. S5 DNA supercoiling in the sco4804 overexpressing strain. DNA supercoiling density of the reporter plasmid pWHM3Hyg isolated from the sco4804 overexpressing MG66 strain derivative (MG66_RP), induced with 10 µg/ml thiostrepton for 45 min or cultured for 24 h in the presence of inducer compared to the non-induced control, the wild-type strain derivative (MS10) and the TopA-depleted strain derivative (MS11) (representative images of two independent replicates are shown). The figure shows topoisomers detected in agarose gel as well as band intensity measurements performed using ImageJ software. [file 12934_2021_1590_MOESM5_ESM.pdf]

Fig. S7

Topoisomerase activity assay

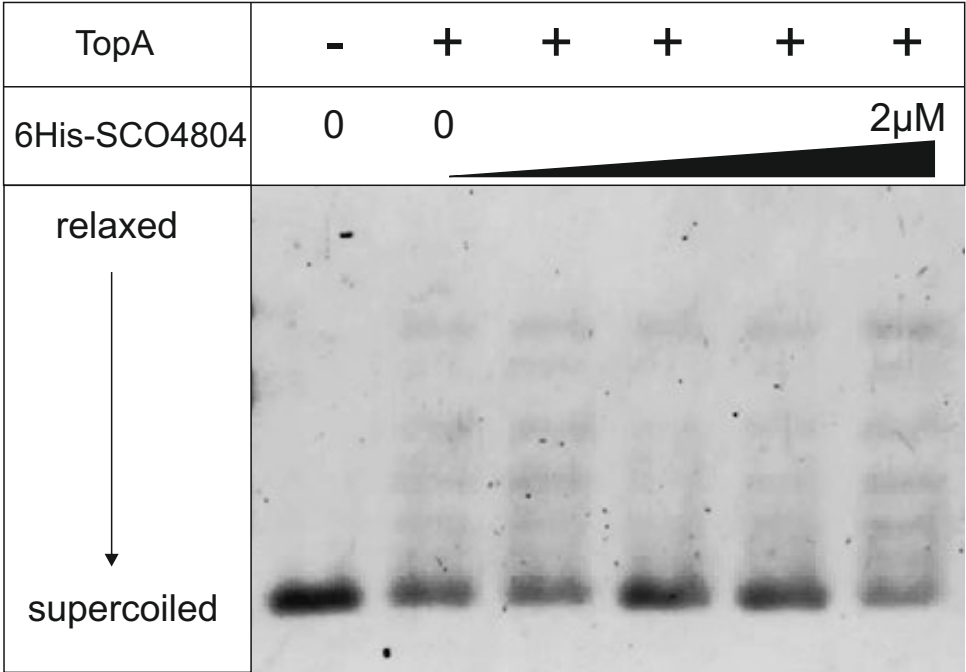

Supplement: Supplementary file 7 — Additional file 7: Fig. S7 Gel electrophoresis demonstrating TopA activity in the presence of 6His-SCO4804 recombinant protein. The assay was performed using 120 ng of TopA and 100 ng of pUC19 plasmid and increasing concentrations of 6His-SCO4804 recombinant protein. The reaction was incubated at 37 °C for 15 min and subsequently stopped by the addition of 2 μl of 0.5 M EDTA. The samples were subsequently resolved on a 0.8% agarose gel in TAE buffer for 14–16 h at low voltage (2 V/cm). [file 12934_2021_1590_MOESM7_ESM.pdf]

Fig. S8

Pull-down experiment

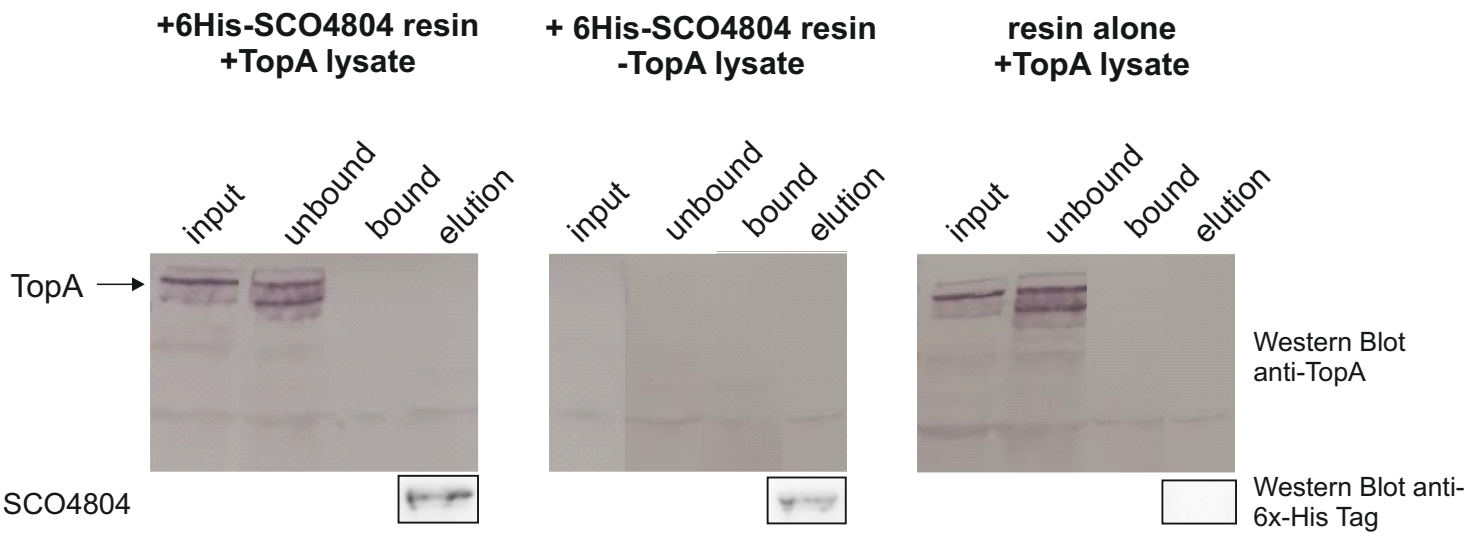

Supplement: Supplementary file 8 — Additional file 8: Fig. S8 Pull-down experiment using 6His-SCO4804 recombinant protein bound to Ni–NTA agarose resin. The resin was subsequently incubated with lysate of the TopA-induced PS04 strain (TopA + lysate). The negative controls served as lysates of the TopA-depleted PS04 (TopA-lysate) strain loaded on 6His-SCO4804 – Ni–NTA agarose and Ni–NTA resin lacking immobilized 6His-SCO4804 recombinant protein. The incubation was performed in TN buffer with 40 mM imidazole. Elution of specifically bound protein was performed using 200 mM imidazole in TN buffer. The samples for the Western blot analysis were prepared using 15 µl of lysate fractions and unbound fraction, 10 µl of eluted proteins and 5 µl of resin as a bound fraction. Samples were resolved using SDS-PAGE and visualized using Western blot and anti-TopA polyclonal antibodies as well as 6xHis tag monoclonal antibody (MA1-135, Thermo Fisher Scientific) to confirm efficient 6His-SCO4804 binding to the resin and its subsequent elution. [file 12934_2021_1590_MOESM8_ESM.pdf]
