# Supplementary material for: Combining transposon mutagenesis and reporter genes to identify novel regulators of the topA promoter in Streptomyces
Source: Microb Cell Fact. 2021 May 13;20:99. doi: 10.1186/s12934-021-01590-7 (PMC8120823; doi:10.1186/s12934-021-01590-7)

# Fig. S6

6His-SCO4804 purification

**A**

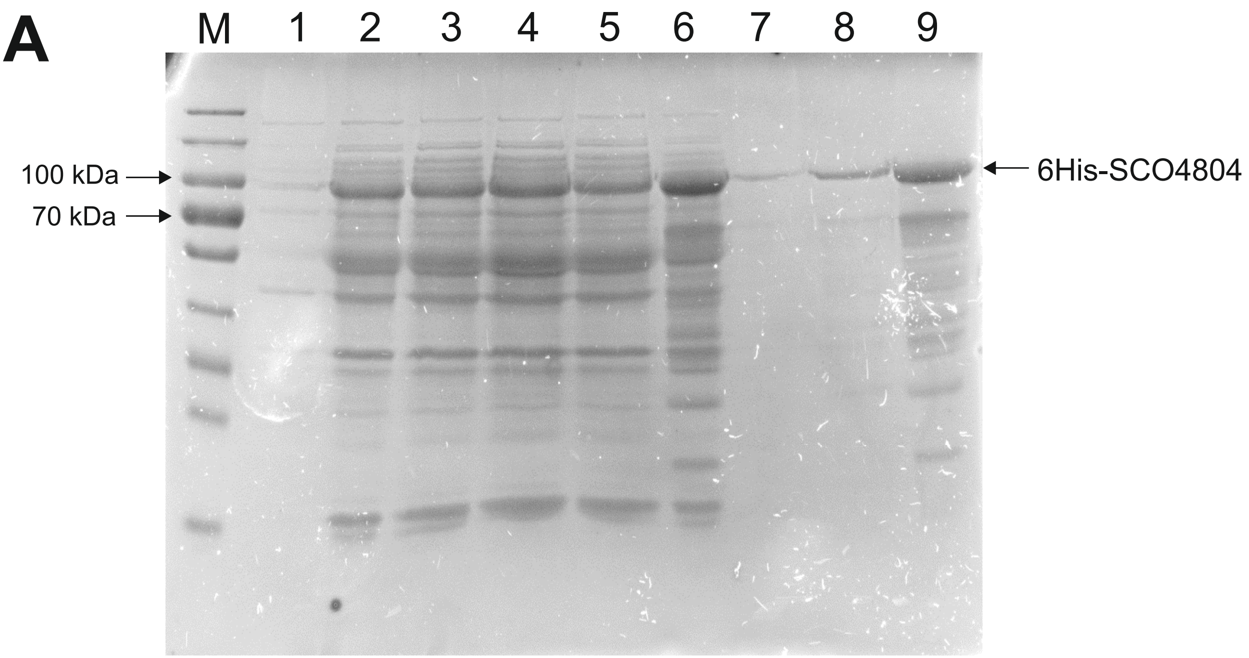


**B**

Electrophoretic mobility shift assay of *topA* promoter and 6His-SCO4804

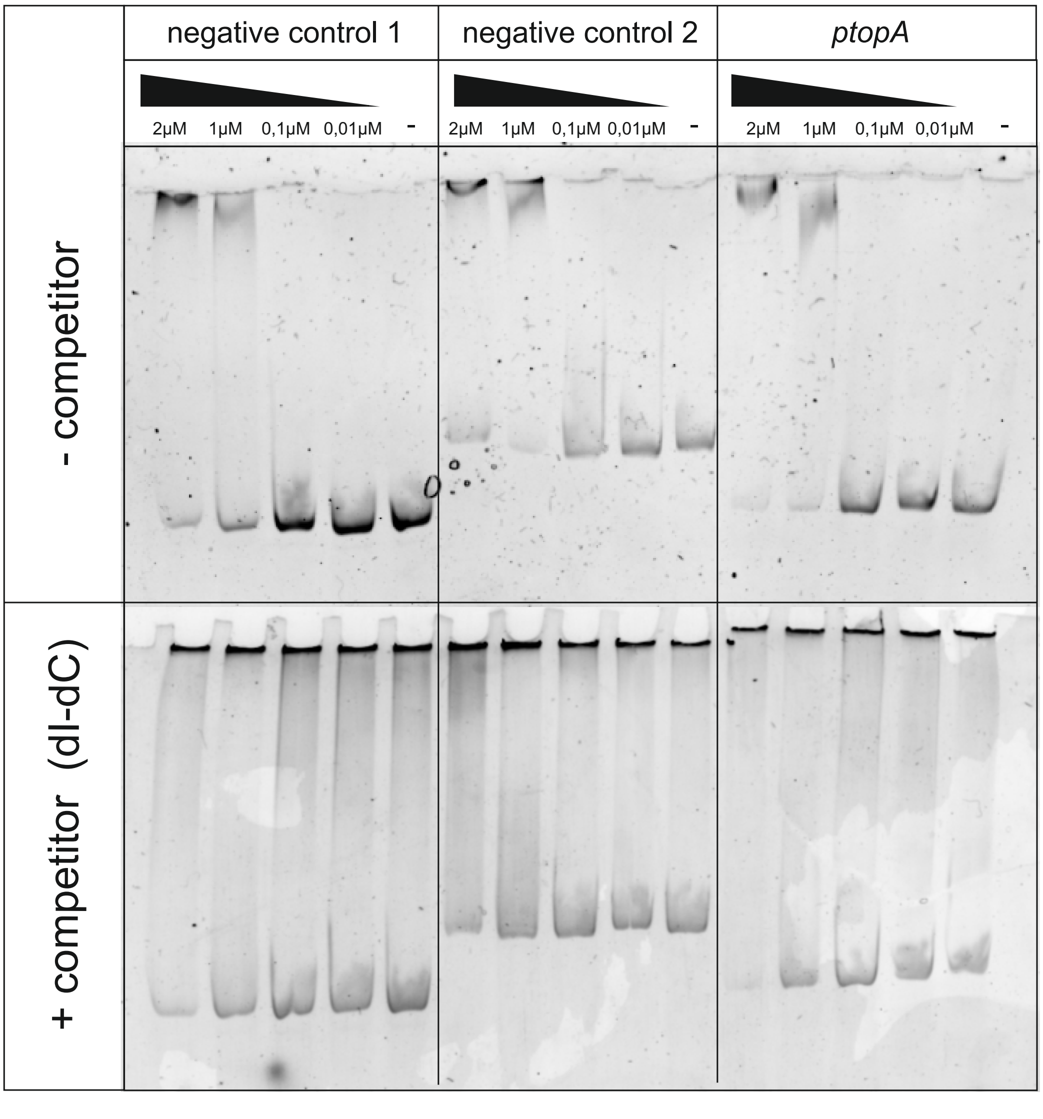

Supplement: Supplementary file 6 — Additional file 6: Fig. S6 Purification of 6His-SCO4804 recombinant protein and DNA binding analysis. A. SDS-PAGE analysis of the collected fractions obtained during 6His-SCO4804 purification from E. coli BL21 (DE3) groEL-groES. M—Molecular mass marker, 1—non-induced E. coli cell extract, 2—induced E. coli cell extract in sarcosyl buffer, 3—induced E. coli cell extract in binding buffer, 4—induced E. coli cell extract in binding buffer, soluble fraction, 5—flow-through, 6—proteins bound to Ni–NTA agarose, 7—wash with binding buffer with 40 mM imidazole, 8—proteins eluted by 200 mM imidazole, 9—the resin after elution. B. Electrophoretic mobility shift assay (EMSA) performed with 30 ng of 461 bp dsDNA fragment of the topA promoter and two negative controls as follows: negative control 1, a 415 bp DNA fragment encompassing the non-coding region between sco4696 and sco4697 genes, and negative control 2, a 654 bp fragment of the sco3928 gene. Binding was performed in PBS containing 5 mg/ml BSA, 5% (v/v) glycerol and, optionally, 2 ng/µl poly(dI-dC). The samples were resolved on a 5% polyacrylamide gel run at 4 °C in 0.25 × Tris–borate-EDTA (TBE) buffer (22.5 mM Tris, 22.5 mM boric acid, 0.5 mM EDTA) at 100 V for 3–4 h. The bands were visualized with ethidium bromide solution that was incubated for 30 min at room temperature and with a ChemiDoc XRS + system (Bio-Rad). [file 12934_2021_1590_MOESM6_ESM.pdf]
