# Supplementary material for: Combining transposon mutagenesis and reporter genes to identify novel regulators of the topA promoter in Streptomyces
Source: Microb Cell Fact. 2021 May 13;20:99. doi: 10.1186/s12934-021-01590-7 (PMC8120823; doi:10.1186/s12934-021-01590-7)
Supplement: Supplementary file 9 — Additional file 9. Detailed informations on heterological overexpression of 6His-SCO4804, in vitro experiments, plasmids and oligonucleotides used in the study. [file 12934_2021_1590_MOESM9_ESM.docx]

**Additional file 9**

**Heterological overexpression of 6His-SCO4804**

For purification of the 6His-SCO4804 protein, the *sco4804* gene was first synthetized with flanking NdeI and EcoRI restriction sites and cloned into the pUCmini vector, yielding pUCmini_*sco4804* (Genscript Biotech Corporation, New Jersey, US). Next, pUCmini_*sco4804* was digested with NdeI and EcoRI, and the 2450-bp fragment containing the *sco4804* gene was cloned into the pET-28a (+) (Novagen) vector digested with the same restriction enzymes, yielding pET28_*sco4804*. To overexpress *6his*-*sco4804,* the *E. coli* strain BL21 (DE3) groEL-groES (Goloubinoff et al., 1989) was transformed with the pET28a_*sco4804* plasmid. For protein overproduction, the *E. coli* BL21 groEL-groES pET28a_*sco4804* strain was grown at 37°C to an optical density at 600 nm (OD_600nm_) of 0.4-0.6; then, cultures were cooled to 20°C, isopropyl-D-thiogalactopyranoside (IPTG) was added to a final concentration of 0.5 mM, and the culture was continued at 20°C overnight. The cells were collected by centrifugation, resuspended in TN buffer (0.05 M Tris pH 8.0, 0.15 M NaCl) supplemented with 1% sarcosyl, sonicated and then the obtained lysate was diluted 125 times with TN buffer containing 2% Triton X-100 to a final concentration of 0.008% sarcosyl (Park et al., 2011). The 6His-SCO4804 protein was then bound to Ni-NTA agarose (Qiagen, Germany) and equilibrated with TN buffer, followed by washing of non-specifically bound proteins with 20- and 40-mM imidazole in TN buffer. The elution of specifically bound proteins was performed using TN buffer with 200 mM imidazole. Proteins that were eluted and bound to the agarose resin were analysed by SDS-PAGE. For 6His-SCO4804 protein visualization, Western blot analysis was performed using 6xHis Tag Antibody (1:500 in TBST, overnight at 4°C; Thermo Fisher Scientific) and horseradish peroxidase-conjugated goat anti-mouse antibodies (1:5000 in TBST, 1 hour at room temperature) (Invitrogen, Carlsbad, CA).

**Topoisomerase activity assay**

Topoisomerase activity was assayed as described earlier (Szafran et al., 2014). Briefly, 200 μl reaction mixtures containing 100 ng of negatively supercoiled pUC19 plasmid, variable amounts of SCO4804 protein and 120 ng of TopA protein (Szafran et al., 2013) were incubated at 37°C for 15 minutes, and then the reaction was stopped by the addition of 2 μl of 0.5 M EDTA. The samples were subsequently resolved by 0.8% agarose gel electrophoresis in TAE buffer for 14–16 hours at low voltage (2 V/cm). The topoisomer distributions were analysed by staining the gel with ethidium bromide/TAE solution at room temperature for 30 min and visualized using ChemiDoc XRS+ (Rio-Rad, CA, US).

**Pull down assay**

For the pull-down assay, the *S. coelicolor* cell lysate and the 6His-SCO4804 protein bound to Ni-NTA agarose (Qiagen, Germany) equilibrated with 40 mM imidazole in TN buffer were used. In parallel, *S. coelicolor* PS04 was cultured in 50 ml of 79 liquid medium supplemented with 10 µg of thiostrepton to induce TopA protein production. The PS04 culture untreated with thiostrepton served as a negative control in the experiment. After 24 hours of growth, the mycelium was collected by centrifugation, resuspended in TN buffer, sonicated and centrifuged to obtain the fraction of soluble *S. coelicolor* proteins. Next, Ni-NTA agarose with 6His-SCO4804 resin was incubated either with the lysate of PS04 culture induced with 10 µg/ml thiostrepton or with TopA-depleted PS04 culture as the negative control. As an additional negative control for the experiment, pure Ni-NTA agarose resin was used and incubated with induced PS04 lysate. After 1 hour of incubation with *S. coelicolor* lysates at 4°C, proteins unbound to 6His-SCO4804-Ni-NTA agarose were collected, and the resin was washed with TN buffer supplemented with 20 and 50 mM imidazole. After washing, 6His-SCO4804 together with potential protein partners was eluted using 200 mM imidazole in TN buffer. Samples for protein gel electrophoresis were prepared using 15 µl of each unbound fraction, 10 µl of eluted proteins and 5 µl of resin. Samples were resolved using SDS-PAGE and visualized by Western blot analysis using anti-TopA and 6xHis Tag antibodies.

**Electrophoretic mobility shift assay (EMSA)**

For EMSA experiments, three dsDNA fragments were amplified using PCR, and the oligonucleotides are listed in Table S1. PCR products were treated with exonuclease I and FastAP Thermosensitive Alkaline Phosphatase (Thermo Fisher Scientific) according to the manufacturer’s instructions and subsequently purified using a DNA Clean-Up kit (A&A Biotechnology). Then, 6His-SCO4804 protein at concentrations up to 2 µM was incubated with 30 ng of dsDNA PCR products for 30 min at 25°C in phosphate-buffered saline (PBS) with 5 mg/ml BSA, 5% glycerol and optionally 2 ng/µl poly(dI-dC). Subsequently, all samples were resolved on a 5% polyacrylamide gel in 0.25× TBE at low voltage for 3-4 hours at 4°C and 100 V. The gel was treated with ethidium bromide solution for 30 min at room temperature, and the DNA bands were visualized using a ChemiDoc XRS+ system (Bio-Rad).

**Table S1: Plasmids used in this study.**

| Name | Description | Reference |
| --- | --- | --- |
| pHL734 | Tn5-based transposon vector; ampicillin and apramycin resistance | Xu et al., 2017 |
| pUC19 | Basic cloning vector that conveys Amp resistance, 2686 base pairs in length, high copy number  pUC19 carries a 54 base-pair multiple cloning site polylinker | Lab stock |
| pUCmini_*sco4804* | pUCmini plasmid containing the *sco4804* newly synthetized gene, flanked by NdeI and EcoRI restriction sites, ampicillin resistance | GeneScript, New Jersey, US |
| pIJ6902 | Chromosomally integrating (φC31) *Streptomyces* *ptipA* expression vector, apramycin and thiostrepton resistance cassettes | Huang et al., 2005 |
| pIJ6902_*sco4804* | pIJ6902 with *sco4804* gene cloned in EcoRI and NdeI sites, apramycin resistance | This study |
| pET28a (+) | Protein expression vector carrying an N-terminal His-Tag/thrombin/T7-Tag configuration plus an optional C-terminal His-Tag sequence, kanamycin resistance | Novagen |
| pET28a_*sco4804* | Plasmid pET28a(+) with *sco4804* gene cloned in EcoRI and NdeI sites | This study |
| pWHM3Hyg | *Streptomyces* supercoiling reporter plasmid, hygromycin resistance | Szafran et al., 2013 |
| pWHM3Spec | *Streptomyces* supercoiling reporter plasmid, spectinomycin resistance | This study |
| pFLUXH*ptopA* | Chromosomally integrating (φBT1) *Streptomyces* vector, *luxCDABE* reporter genes under control of *topA* promoter, hygromycin resistance cassettes | Szafran et al., 2016 |

**Table S2: Oligonucleotides used in this study.**

| Oligonucleotide | Sequence | Description |
| --- | --- | --- |
| UpS | GAGTTAGCTCACTCATTAGGC | Identification of Tn5 insertion site |
| M13pUC_RV | agcggataacaatttcacacagg | Verification of pIJ6902_4804 plasmid |
| SCO4804_RV | GGTGCTCGATCACGTCCTC |  |
| RT3543_FWD | ACGACTTCCAGCCGATCTATGT | RT-qPCR analysis of *topA* gene |
| RT3543_RV | GGAACACCATGCGCTTGAC |  |
| hrdBRT_F | TGCTCTTCCTGGACCTCATC | RT-qPCR analysis of *hrdB* gene |
| *hrdB*RT_R | GTAGCCCTTGGTGTAGTCGAA |  |
| RTPCR3874FW | TCGACTTCGTGACGTACCTCAA | RT-qPCR analysis of *gyrB* gene |
| RTPCR3874RV | TCGAGGGACAGGTCTTGTC |  |
| RT_4804_FW | GGGGCTGCCCAGGACCA | RT-qPCR analysis of *sco4804* gene |
| RT_4804_RV | GCCTGCGATGGGTCCAGG |  |
| RT_4805_FW | CGTTCGGTCAGGAAGGATATCG | RT-qPCR analysis of *sco4805* gene |
| RT_4805_RV | TACACGAGGGAAGCCGCCAT |  |
| luxC_RT_FW | CGGCGACAACAGCGTCTA | RT-qPCR analysis of *luxC* gene |
| luxC_RT_RV | CAACCGCAGCTTGTTGTTCTC |  |
| 4696_FW | ccggatccgaggattcgccttcaccgcg | EMSA dsDNA fragments amplification |
| 4697_RV | ggcatatgcgatacccccgtcggctc |  |
| 3928_FLANK_FW | GTAATGCCGGCGTAGGGAG |  |
| SEQ_3928_RV | CTTCACCAGGAACCGCTTG |  |
| topA_prom_FW | GCGGATCCGATCTTCGCGGGAGTGGTGTTC |  |
| topA_prom_RV | CGCATATGCGCTCTTCTCTCCGGTCGACGC |  |
